# Successful repair of recurrent rectovaginal fistula by stratified suture using transanal endoscopic microsurgery: A CARE-compliant case report

**DOI:** 10.1097/MD.0000000000004600

**Published:** 2016-09-09

**Authors:** Weijie Chen, Xin Chen, Guole Lin, Huizhong Qiu

**Affiliations:** a Department of Surgery; b Department of Orthopedics, Peking Union Medical College Hospital, Chinese Academy of Medical Sciences, Shuaifuyuan, Beijing, P. R. China.

**Keywords:** case report, rectovaginal fistula, surgery, transanal endoscopic microsurgery

## Abstract

**Background:**

Rectovaginal fistulas (RVFs) are abnormal connections between the rectum and vagina. Although many surgical approaches to correct them have been attempted, management of RVFs still remains a challenge, especially for recurrent RVFs.

**Methods:**

In the present study, we report a case in a 22-year-old female with a chief complaint of obvious passages of flatus or stool through the vagina for 10 years. She had suffered a vaginal trauma from a violent accident 10 years prior, and gradually noticed the uncontrollable passage of gas or feces from the vagina 2 weeks later.

The patient underwent a transvaginal direct repair surgery at local hospital 9 years ago, but the symptoms recurred 1 month after the surgery. After 2-years monitoring, the patient underwent another transvaginal repair surgery (fistulectomy followed by direct suture) at another hospital, but the fistula recurred again. We initially performed a temporary protective transversostomy upon admission. After 8-months of observation, a methylene blue test was conducted and the diagnosis of recurrent RVF was confirmed. Subsequently, we performed a successful repair by stratified suture using transanal endoscopic microsurgery (TEM). The scar tissue on the posterior wall of the vagina and the anterior wall of the rectum were meticulously excised until the margin of the excisional line showed healthy tissue. In addition, the fistulous tract was completely removed. The edges of the fistula on the posterior wall of the vagina were closed by simple continuous suturing, and the rectal anterior wall was sutured in the same manner.

**Results:**

During a 1-year follow-up period, the fistulae were not recurrent and no complication such as incontinences or rectal bleeding were found. The latest Wexner score was 3.

**Conclusion:**

We present a case of successful treatment with stratified suture using TEM throughout the procedure. We strongly recommend this efficient and minimally invasive procedure for recurrent RVFs.

## 1 Introduction

Rectovaginal fistulas (RVFs) are abnormal connections between the rectum and vagina, which lead to leakage of rectal contents through the vagina. Common factors contributing to their formation are trauma (mostly resulting from obstetric surgeries), inflammatory bowel disease, infection, tumor, and prior history of pelvic radiation.^[1]^ Multiple surgical procedures have been attempted, ranging from direct repair, plug placement, or advancement flap for smaller defects, to muscle interposition or laparotomy for larger defects. In addition, colostomy, proctectomy, or delayed pull-through coloanal anastomosis are suggested for complex RVFs.^[2]^

However, management of RVFs remains a challenge, especially for recurrent RVFs. Regardless of the surgical option chosen, the failure rate for repairs and the recurrence rate of RVF were high.^[3]^ Recurrent fistulae are considered more complex due to their association with tissue scarring and decreased blood supply.^[2]^ The success rate decreases with each additional repair attempt. The first attempt success rate of RVF repair with a mucosal advancement flap is 88%, but this decreases to 85% after a previous repair, and 45% to 55% with 2 previous attempts.^[4]^ Patients with RVFs bear an enormous emotional, psychological, and social burden. Such a problem might be particularly distressing in cases of repeatedly failed surgeries. In the current study, we report a case of recurrent RVF, who underwent 2 failed previous repairs elsewhere, and for which we performed a successful repair by stratified suture using transanal endoscopic microsurgery (TEM).

## 2 Case report

A 22-year-old Chinese female was admitted to our hospital with a chief complaint of obvious passages of flatus or stool through the vagina for 10 years. She had suffered a trauma of the vagina from a violent accident 10 years prior, and 2 weeks later, she gradually noticed uncontrollable passage of gas or feces from the vagina, with more subtle presentations being slight fecal discharge. The symptoms, however, were more prominent when feces appeared in liquid form, especially when she had episodes of diarrhea. She denied fever, abnormal vaginal discharge, leakage of urine, rectal bleeding, rectal tenesmus, fecal incontinence, urinary incontinence, anus bulge, and pain at that time. She had received no surgery previously and her menses did not commence upon initial clinical presentation. The patient was diagnosed with RVF, and had undergone a transvaginal direct repair surgery at a local hospital 9 years prior. However, the symptoms recurred 1 month after the 1st surgery. After 2-years of monitoring, the patient underwent another transvaginal repair at a different hospital. A fistulectomy followed by direct suture was performed by a gynecologist, but the fistula recurred 2 weeks later. Following this surgery, the patient had not received any other surgery prior to admission to our hospital. She is a nonsmoker. In addition, we did not identify any special circumstances regarding her previous history or family history relevant to her presentation. Upon physical examination, she was afebrile with a blood pressure of 125/80 mm Hg and a regular pulse of 70 bpm. Her abdomen was soft without tenderness. Bowel sounds were normal at 3 to 5/minute. Digital rectal examination and rigid sigmoidoscopy revealed a lesion approximately 4 cm from the anal verge at 6 o’clock in the knee-chest position. The diameter of the lesion was approximately 1 cm. No abscess, sepsis, or fecal incontinences were found.

We first performed a protective transversostomy. After 8-months observation, a methylene blue test was conducted in which the rectal cavity was irrigated with approximately 20 mL of methylene blue solution and a sterile gauze that was placed in the vagina was stained blue indicating a persistent fistula. Hence, the diagnosis of recurrent RVF was confirmed. An additional intervention was warranted, and the patient was re-admitted to our hospital.

**Figure 1:**
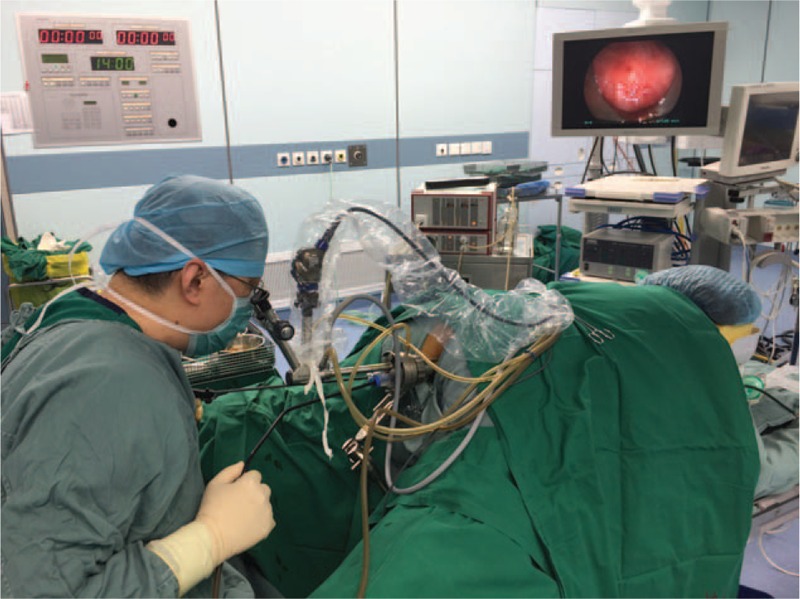
An intraoperative photograph taken during rectovaginal fistulae repair with transanal endoscopic microsurgery (TEM).

**Figure 2:**
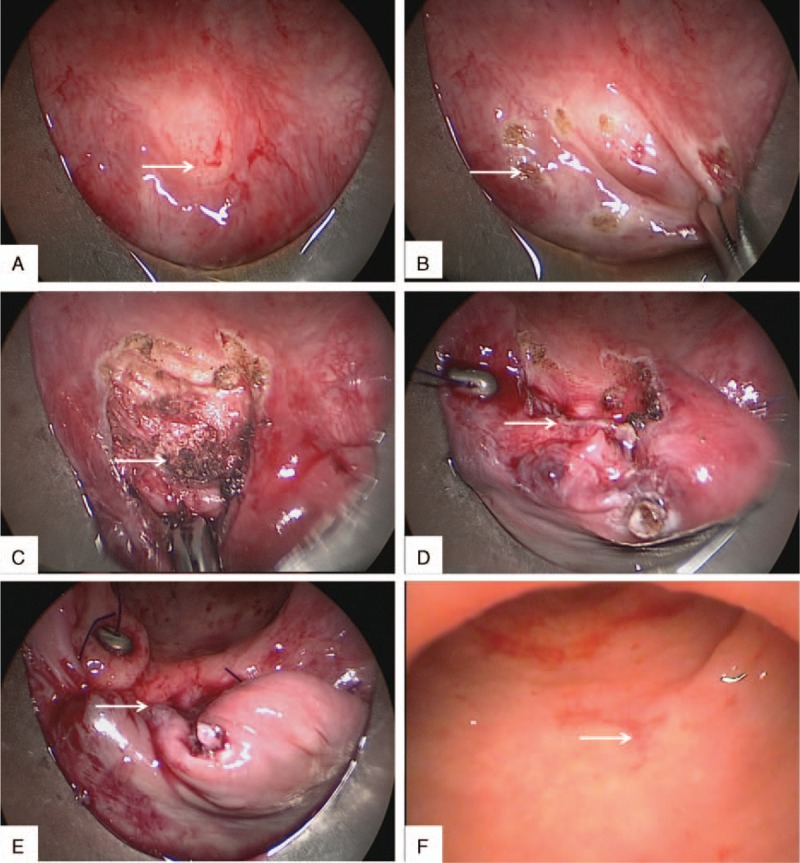
Stratified suture to repair recurrent rectovaginal fistulae using transanal endoscopic microsurgery (TEM). (A) The endoscopic image before repair. The arrow shows the orifice of the recurrent rectovaginal fistulae. (B) Marking the resection area with coagulation dots using a needle electrode before resection. The arrow indicates the dots. (C) The vaginal muscular layer was exposed after the excision of the scar tissue and sclerotic fistula tissue. The arrow indicates the vaginal muscular layer. (D) The vaginal muscular edge was sutured with absorbable suture. The arrow indicates the suture line of the vaginal muscular layer. (E) The full-thickness layer of the rectal posterior wall was sutured. The arrow indicates the suture line of the rectal layer. (F) The endoscopic image 6 months after surgery. The arrow indicates the scar of the rectum.

We performed a stratified suture to repair the fistula using TEM (Richard Wolf GmbH, Knittlingen, Germany) under general anesthesia. Preoperative chemical preparation was achieved by administration of 1.5 g cefoperazone and 1 g metronidazole 15 minutes prior to operation. No preoperative mechanical bowel preparation (e.g., enema) was required due to previous colostomy. The patient was positioned in the prone position. A 40 mm diameter rectoscope was inserted into the anus after gentle dilation. Afterwards, the orifice was exposed under direct vision (Fig. 1). A 0.5 to 1 cm resection margin was marked around the lesion with a needle diathermy (Fig. 2). The scar tissue on the posterior wall of the vagina and the anterior wall of the rectum were meticulously excised until the margin of the excisional line showed healthy tissue. In addition, the fistulous tract was completely removed. After irrigating the posterior wall with copious normal saline, the edges on the posterior wall of the vagina were closed by simple continuous suturing with 3/0 absorbable monofilaments oriented longitudinal to the vaginal wall. The hemostasis was carefully verified, and the full-thickness layer of the rectal anterior wall was sutured in the same direction. The rectal wall, not the 2nd suture line, should cover the previous suture line. The operation time was approximately 40 minutes with blood loss of 10 mL.

The patient did not complain of any discomfort such as pain, flatulence, or rectal bleeding after the surgery and an elementary diet was initiated on the 2nd day. No analgesics or antibiotics were required postoperatively. The patient was discharged 2 days after the operation. Radiologic evaluation performed on postoperative day 10 showed no fistulas. At postoperative month 6, the transversostomy was closed. During a 1-year follow-up, the fistula was not recurrent and no complication of TEM, such as incontinences or rectal bleeding, was found. There was no significant difference in bowel evacuation habits before and after surgery (1–2 times per day). The latest Wexner score was 3.

The study was approved by the ethics committee of Perking Union Medical College Hospital, and informed consent was received from the patient.

## 3 Discussion

Recurrent RVFs are difficult to manage. After an initial failed attempt, a simple fistula becomes complex. The poor viability of the surrounding tissues, the presence of inflammation, infection or scar tissue, and the inappropriate choice of surgical repair may lead to failure of subsequent surgical procedures. Logically, local repairs are reasonable to attempt initially because they are less invasive. However, the anovaginal septum is thin and poorly vascularized. Local closures with advancement flaps or biomaterials have been reported to be associated with relatively high recurrence rates, most likely due to inadequate well-vascularized tissue bulk.^[4,5]^ Reconstruction through interposition of autologous tissues (Martius flap, gracilis muscle) seems more promising because introduction of healthy vascularized tissues creates better conditions for healing. Nevertheless, postoperative complications such as temporary leaks, vaginal sepsis, partial skin paddle necrosis, or vaginal stricture are not uncommon.^[6]^ In addition, these reconstruction methods appear to be more invasive and might prolong in-hospital stays. There is promising evidence that complex high RVFs can be managed through abdominal approaches.^[7]^ Such approaches, however, are relatively technical demanding and rather aggressive. We propose that these approaches should not be a first choice for recurrent RVFs, but their value in complex scenario where local repairs repeatedly fail should not be overlooked. Although many types of procedures have been reported in the literature, there is no standard treatment strategy for the management of RVFs.^[8]^ The choice of surgical approaches has primarily been determined by the surgeon's own judgment and based on heterogeneous presentations, etiologies, and surrounding tissue conditions.

There is still controversy over the role of protective ostomy in RVF repairs. Lambertz et al^[9]^ reported that no differences were found regarding fistula recurrence rates between those who received stoma and those without, and patients treated with protective stoma had significantly longer in-hospital stays. However, Corte et al^[3]^ claimed that the repair success rate of RVF patients who underwent procedures with stoma (32%) was significantly higher than those without stoma (6%). To optimize outcomes, it is important to ensure that no sepsis or inflammation exists before making local repairs especially in complex RVFs. Fecal diversion would help resolve any active inflammation and allow the tissue to soften. The inflammation in the rectal tissue of our patient was severe, and a great deal of scar tissue existed. Thus, we performed a protective ostomy and then a stratified suture using TEM.

TEM is a safe and feasible minimally invasive surgical approach to treat lesions of the mid and lower rectum. It was introduced into clinical practice in 1983 and was originally intended for the treatment of rectal adenomas and early stage cancer.^[10]^ After decades of development, the procedure has become more advanced. With the advantage of full-layer suturing under proctoscopy, TEM can address most perforations encountered in an operation, which is a hazardous complication of localized rectum resection. TEM was first reported for the repair of simple RVF in 2008.^[11]^ It provides a magnified optimal visual view of up to 6-fold and a broadened operative field by carbon dioxide insufflations. With these advantages, we thought this approach could also be applied in the repair of recurrent RVFs. The TEM procedure enables the accurate determination of anatomical structure as well as the identification of the fistulous tract. The scar and sclerotic tissue as well as the epithelium overlying the fistulous tract, which we believe to be one of the culprits responsible for recurrences, can accurately be removed by needle diathermy without significant loss of the normal tissue. As a result, the optimal vascularization and decrease in tension between the sutured edges might promote healing. The 2 previous recurrences of our patient might be due to insufficient exposure of anatomical structures using transvaginal repairs. Second, it is also necessary for each layer to be sutured in a stratified fashion to withstand the higher pressure coming from the rectal side.^[12]^ It is noteworthy that we intentionally closed suture lines at different positions, nonoverlapping positions to increase the durability of the repair. The transverse direction of the suture line avoids postoperative anal stricture. Moreover, the procedure has the advantage of minimal invasiveness. It avoids any incision in the perineal area, which can be painful and is probably detrimental to sphincter function. In addition, the obvious advantages of shorter operative time, less intraoperative bleeding, reduced in-hospital stays, and significantly less postoperative complications make TEM a safe and effective measure for the repair of complex RVFs in selected patients and potentially will make it a “first-line choice” in the future.

We are also aware of the previous work by D’Ambrosio on the treatment of RVFs with TEM.^[13]^ However, we noticed their procedure calls for a blind dissection of the rectovaginal septum using a finger. Blind manual dissection could cause injury to the surrounding healthy tissue. Tearing of the mucosa or muscular layer may be responsible for complications such as hematoma and abscess formation reported in the literature. In our case, on the other hand, we took full advantage of the TEM magnified visual system and were able to complete the entire surgical procedure under rectoscopy. The precise dissection of the fistula enabled us to preserve the surrounding healthy tissue and decrease tension between the edges used for suturing. In addition, we employed stratified suturing to minimize the space between the different layers, thus reducing the likelihood of the formation of a hematoma or abscess. Thus far, TEM has played a promising role in the treatment of recurrent RVFs especially from traumatic etiology. Long-term outcomes still need to be determined using a larger series of patients. Additionally, the efficacy of TEM in the treatment of recurrent RVFs in patients receiving radiotherapy, accompanied by rectal cancer or with high RVFs, needs to be determined.

We present a successful case of treatment with stratified suture using TEM throughout the procedure. We strongly recommend this efficient and minimally invasive procedure.
